# CDKN2A/B co-deletion is associated with increased risk of local and distant intracranial recurrence after surgical resection of brain metastases

**DOI:** 10.1093/noajnl/vdad007

**Published:** 2023-01-28

**Authors:** Ramin A Morshed, Minh P Nguyen, Daniel D Cummins, Satvir Saggi, Jacob S Young, Alexander F Haddad, Ezequiel Goldschmidt, Edward F Chang, Michael W McDermott, Mitchel S Berger, Philip V Theodosopoulos, Shawn L Hervey-Jumper, Mariza Daras, Manish K Aghi

**Affiliations:** Department of Neurological Surgery, University of California, San Francisco, San Francisco, CA, USA; Department of Neurological Surgery, University of California, San Francisco, San Francisco, CA, USA; Department of Neurological Surgery, University of California, San Francisco, San Francisco, CA, USA; Department of Neurological Surgery, University of California, San Francisco, San Francisco, CA, USA; Department of Neurological Surgery, University of California, San Francisco, San Francisco, CA, USA; Department of Neurological Surgery, University of California, San Francisco, San Francisco, CA, USA; Department of Neurological Surgery, University of California, San Francisco, San Francisco, CA, USA; Department of Neurological Surgery, University of California, San Francisco, San Francisco, CA, USA; Division of Neurosurgery, Miami Neuroscience Institute, Miami, FL, USA; Department of Neurological Surgery, University of California, San Francisco, San Francisco, CA, USA; Department of Neurological Surgery, University of California, San Francisco, San Francisco, CA, USA; Department of Neurological Surgery, University of California, San Francisco, San Francisco, CA, USA; Department of Neurological Surgery, University of California, San Francisco, San Francisco, CA, USA; Department of Neurological Surgery, University of California, San Francisco, San Francisco, CA, USA

**Keywords:** brain metastasis, CDKN2A, CDKN2B, distant recurrence, local recurrence, surgery

## Abstract

**Background:**

While genetic alterations in brain metastases (BMs) have been previously explored, there are limited data examining their association with recurrence after surgical resection. This study aimed to identify genetic alterations within BMs associated with CNS recurrence after surgery across multiple cancer types.

**Methods:**

A retrospective, single-center study was conducted with patients who underwent resection of a BM with available clinical and gene sequencing data available. Local and remote CNS recurrence were the primary study outcomes. Next-generation sequencing of the coding regions in over 500 oncogenes was performed in brain metastasis specimens. Cox proportional hazards analyses were performed to identify clinical features and genomic alterations associated with CNS recurrence.

**Results:**

A total of 90 patients undergoing resection of 91 BMs composed the cohort. Genes most frequently mutated in the cohort included *TP53* (64%), *CDKN2A* (37%), *TERT* (29%), *CDKN2B* (23%), *NF1* (14%), *KRAS* (14%), and *PTEN* (13%), all of which occurred across multiple cancer types. *CDKN2A/B* co-deletion was seen in 21 (23.1%) brain metastases across multiple cancer types. In multivariate Cox proportional hazard analyses including patient, tumor, and treatment factors, *CDKN2A/B* co-deletion in the brain metastasis was associated with increased risk of local (HR 4.07, 95% CI 1.32-12.54, *P* = 0.014) and remote (HR 2.28, 95% CI 1.11-4.69, *P* = 0.025) CNS progression. Median survival and length of follow-up were not different based on *CDKN2A/B* mutation status.

**Conclusions:**

*CDKN2A/B* co-deletion detected in BMs is associated with increased CNS recurrence after surgical resection. Additional work is needed to determine whether more aggressive treatment in patients with this mutation may improve outcomes.

**Key Points**

- *CDKN2A/B* co-deletion is frequently found in brain metastases across multiple cancer types
- *CDKN2A/B* co-deletion is associated with local and distant CNS recurrence of brain metastases after surgical resection

**Importance of Study**

Brain metastases are frequently resected to achieve local CNS control providing the opportunity to correlate genomic alterations with surgical outcomes. Prior work has examined genomic alterations associated with metastatic spread to the CNS. However, there is a paucity of data correlating specific gene mutations with clinical outcomes in surgical cohorts of patients with brain metastases. This study aimed to evaluate genomic alterations in surgically resected brain metastases across various cancer types and identify alterations associated with an increased risk of CNS recurrence after surgical resection. Patients harboring *CDKN2A/B* co-deletion in resected brain metastases were at increased risk of both local and distant CNS progression despite similar survival and follow-up duration suggesting a potential biologic role in CNS seeding and progression. To our knowledge, this is the first time, this genetic alteration has been reported to be associated with surgical outcomes after brain metastasis resection.

Brain metastases are the most common intracranial malignancy in adult patients, and their incidence is thought to be rising due to improved diagnostic techniques and control of extracranial disease. Despite recently approved novel systemic agents which offer promising control of central nervous system (CNS) disease,^1–4^ control of brain metastases remains a therapeutic challenge. Local control may be achieved with surgical resection of a brain metastasis via a craniotomy, and either preoperative radiosurgery or adjuvant postoperative radiation therapy has been shown to decrease rates of local recurrence.^5–10^ However, the risk of local and distant CNS recurrence after surgery is still relatively high,^11,12^ and factors predicting recurrence are not well defined.

Brain metastases are frequently resected as part of clinical care for local control purposes, and this tissue provides the opportunity for insights into genomic alterations commonly seen in brain metastases across various cancer types and correlation with surgical outcomes. Prior work has examined gene signatures and genomic alterations associated with metastases forming within the brain.^13–15^ However, there is a paucity of data correlating specific gene mutations with clinical outcomes in surgical cohorts of patients with brain metastases. This study aimed to evaluate gene mutations in surgically resected brain metastases across various cancer types as well as identify frequently mutated genes associated with increased risk of CNS recurrence after resection.

## Methods

### Study Design

This was a retrospective cohort study conducted at an academic medical center. After obtaining approval from the UCSF IRB (Study Number 15-17500), the UCSF tumor registry was searched for adult patients who underwent surgical resection of an intracranial brain metastasis between 2014 and 2021. Inclusion criteria were patients who (a) were 18 years old or greater at the time of surgery, (b) underwent a craniotomy for resection of a brain metastasis, (c) had pathology-confirmed malignant tissue present at the time of BM resection, (d) had available gene sequencing data available for the coding regions in over 500 oncogenes, and (e) had an electronic medical record with available imaging and documentation of outcomes clinical outcomes for greater than 1 month. Patients were excluded if they did not have gene sequencing performed. Surgical resection was considered after multidisciplinary discussion between a neurosurgeon, radiation oncologist, and oncologist. The IRB waived the requirement for written informed consent for this retrospective observational study.

### Patient, Tumor, and Treatment Variables

Patient variables included age at surgery, sex, race/ethnicity, minority status, preoperative Karnofsky Performance Scale (KPS) score, and date of death. Tumor variables included primary cancer type, tumor location, tumor side, tumor volume (estimated using the (length × width × height)/2 method previously validated for assessing BM volume^2^), total number of brain metastases at the time of surgery, intratumoral hemorrhage on preoperative imaging, cystic appearance on preoperative imaging, and the presence or absence of extracranial disease at the time of surgery. Extracranial malignant disease status was based on results from either total body positron emission tomography (PET) imaging or computerized tomography (CT) imaging of the body with and without contrast performed for staging purposes obtained within 1 month of the surgery date. Treatment variables included extent of resection (gross total resection (GTR) vs subtotal resection (STR)), number of BMs resected at index surgery, prior radiation to the index brain metastasis (i.e. progressive BMs), and type of postoperative local radiation therapy. A GTR was defined as complete resection of contrast-enhancing tumor on a postoperative MRI scan whereas STR was defined as any residual enhancing mass on the postoperative scan.

### Clinical Outcomes of Interest

The main outcomes of the study were local and distant (i.e. remote) CNS progression after surgical resection. Local recurrence involved new enhancing disease around the resection cavity that demonstrated growth and was not concerning for radiation necrosis. Distant recurrence involved new enhancing lesions remote from the resection cavity or evidence of leptomeningeal disease. Diagnosis was confirmed on imaging with agreement of both neuroradiologists and treating oncologists. If an event was not documented, last clinical follow-up was used for censoring. Follow-up imaging consisted of MRI obtained at intervals at the discretion of the treating oncologist, radiation oncologist, and/or neurosurgeon and were typically in the range of every 3-6 months.

### Statistical Analysis

Statistical analyses were performed in JMP Pro (version 16.0, SAS Institute Inc.) Demographic data and baseline characteristics were assembled and analyzed in a standard fashion. The Kaplan–Meier method was used to visualize time to local and distant CNS progression from surgery and survival from surgery. Univariate and multivariate nominal logistic regression and Cox proportional hazards analyses were performed to identify variables associated with local and distant progression. Odds ratios (nominal regression) and hazard ratios (Cox proportional hazards model) were computed using a 95% confidence interval. Multivariate regression analyses were performed with variables carrying *P* < 0.05 on univariate analysis. The level of significance was 0.05 for all analyses.

## Results

### Clinical Cohort and Outcomes

The cohort consisted of 90 patients undergoing resection of 91 brain metastases with genomic data available. There was 1 patient who underwent resection of 2 separate brain metastases during two separate surgeries 10 months apart. Details of the cohort can be found in **Table 1**. The median age at surgery was 67.2 years, and the cohort consisted of 41 (45.1%) males and 50 (54.9%) females. Median KPS was 80 (range 50-100). The most common primary cancer types were non-small cell lung cancer (*n* = 22, 24.2%), melanoma (*n* = 22, 24.2%), and breast adenocarcinoma (*n* = 15, 16.5%). The most common tumor locations were within the frontal lobe (*n* = 26, 28.6%), parietal lobe (*n* = 24, 26.4%), and cerebellum (*n* = 14, 15.4%). The median number of total brain metastases at the time of index surgery was 2 (range 1-25) and extracranial disease was present at the time of index surgery in 60 (65.9%) cases. The median tumor volume of the resected BM was 15.5 cm^3^ and ranged from 0.3 to 109.9 cm^3^.

**Table 1.** Patient, Tumor, and Treatment Details of the Cohort

Patients	90
Tumors resected with mutation data	91
Age (yrs, range)	67.2 (27.1-84.9)
Male	41 (45.1%)
Female	50 (54.9%)
Median preop KPS (range)	80 (50-100)
Minority	66 (72.5%)
Non-minority	25 (27.5%)
Primary cancer	
NSCLC	22 (24.2%)
Melanoma	22 (24.2%)
Breast	15 (16.5%)
GI	12 (13.2%)
Gyn	4 (4.4%)
RCC	4 (4.4%)
Other	12 (13.2%)
No. total brain mets at surgery	2 (1-25)
Tumor volume	15.5 (0.3-109.9)
Cystic	18 (19.8%)
Non-cystic	73 (80.2%)
Hemorrhage	47 (51.6%)
No hemorrhage	44 (48.4%)
Time from BM diagnosis to surgery (mo, range)	0.3 (0-42.9)
Presence of extracranial disease	60 (65.9%)
Absence of extracranial disease	31 (34.1%)
Side	
Left	43 (47.3%)
Right	45 (49.5%)
Midline	3 (3.3%)
Location	
Frontal	26 (28.6%)
Parietal	24 (26.4%)
Temporal	16 (17.6%)
Occipital	11 (12.1%)
Cerebellum	14 (15.4%)
Extent of resection	
GTR	77 (84.6%)
STR	14 (15.4%)
Prior intracranial radiotherapy	
Yes	16 (17.6%)
No	75 (82.4%)
Postoperative radiotherapy type	
Local RT	74 (81.3%)
WBRT	3 (3.3%)
None	14 (15.4%)
Postoperative checkpoint inhibitor therapy	30 (33%)
Postoperative targeted inhibitor therapy	38 (41.8%)
Local CNS progression	13 (14.3%)
Distant CNS progression	42 (46.2%)
Death	33 (36.3%)
Median length of follow-up (mo, range)	10.3 (0.4-90.2)

Abbreviations: NSCLC, non-small cell lung cancer; GI, gastrointestinal; Gyn, gynecologic; RCC, renal cell carcinoma; GTR, gross total resection; STR, subtotal resection; CNS, central nervous system; RT, radiation therapy; WBRT, whole brain radiation therapy; KPS, Karnofsky Performance Scale.

Treatment details for the cohort are displayed in **Table 1**. A GTR and STR were performed for 77 (84.6%) and 14 (15.4%) of BMs, respectively. Prior intracranial radiation had been used in 16 (17.6%) cases, and postoperative adjuvant radiotherapy was used in 84.6% of the cohort with 74 patients undergoing focal radiation therapy (SRS, SRT, or brachytherapy) and 3 (3.3%) undergoing WBRT. Targeted therapy was used postoperatively in 38 patients (41.8%), and in 22 patients (24.2% of cohort), genomic alterations identified in the resected brain metastasis led to implementation or a change in targeted therapy (**Table 1 in the Supplement**). Checkpoint inhibitor therapy was used postoperatively in 30 patients (33%).

Median follow-up was 10.3 months (range 0.4-90.2). Local CNS progression at the site of prior surgery was observed in 13 patients (14.3% of cohort) with median censored time to local CNS progression not reached. Distant CNS progression remote from the site of index surgery was observed in 42 patients (46.2% of cohort) with median censored time to distant CNS progression found to be 12.5 mo. By the last follow-up, 33 patients had died (36.3% of cohort) and median survival from surgery was not reached.

### Frequent Gene Mutations in Brain Metastases

**Figure 1.**
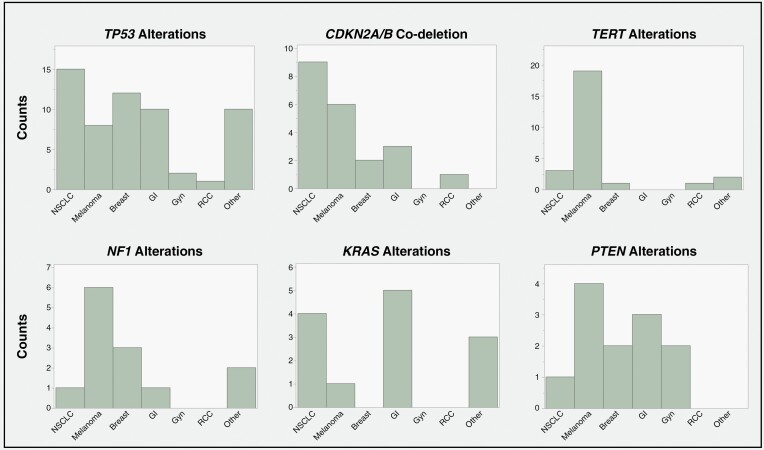
Genes frequently altered across multiple cancer types.

Next-generation sequencing of surgically resected brain metastases was performed by screening the full coding region of over 500 oncogenes. An oncoplot with these results is displayed in **Figure 1 in the Supplement**. The most frequent gene mutations seen in the cohort included *TP53* (64% of specimens), *CDKN2A* (37%), *TERT* (29%), *CDKN2B* (23%), *NF1* (14%), *KRAS* (14%), *PTEN* (13%), and *NRAS* (11%). All of these alterations (except *NRAS* mutations which only occurred within melanoma brain metastases) occurred across multiple cancer types (**Figure 1**). *CDKN2A/B* co-deletion was seen in 21 BMs (23.1% of cohort) across multiple cancer types including NSCLC (*n* = 9, 49.9%), melanoma (*n* = 6, 27.3%), breast (*n* = 2, 13.3%), gastrointestinal (*n* = 3, 25%), and renal cell carcinoma (*n* = 1, 25%) (**Figure 1**).

### CDKN2A/B Co-deletion is Associated with Increased Risk of Local and Distant CNS Recurrence

**Figure 2.**
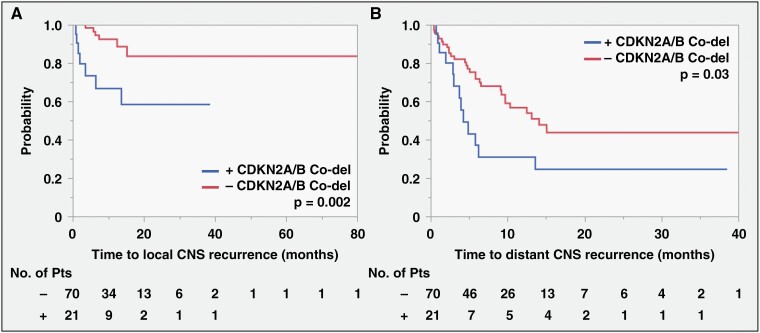
*CDKN2A/B* homozygous co-deletion is associated with decreased time to local and distant CNS recurrence after surgical resection of brain metastases. **(A)** Decreased time to local recurrence was observed for tumor harboring *CDKN2A/B* co-deletion (median time not reached for either group, log-rank *P* = 0.002). **(B)** Decreased time to distant recurrence was observed for tumor harboring *CDKN2A/B* co-deletion (+ vs −co-deletion: 4.2 vs 14.1 mo, log-rank *P* = 0.03).

Commonly mutated genes were then screened for their association with CNS recurrence. *CDKN2A/B* co-deletion was found to be significantly associated with decreased time to local recurrence (**Figure 2A**, log-rank *P* = 0.002). Univariate and multivariate Cox proportional hazard analyses were performed examining patient, tumor, treatment, and genetic factors associated with local recurrence (**Table 2**). *CDKN2A/B* co-deletion and increased time from BM diagnosis to surgery were associated with decreased time to local recurrence on univariate analysis. On multivariate analysis, only *CDKN2A/B* co-deletion (HR 4.07, 95% CI 1.32-12.54, *P* = 0.014) was associated with local CNS recurrence. This relationship was still significant when controlling for primary cancer type, treatment with postoperative adjuvant, prior radiation treatment, and treatment with postoperative systemic therapy in a separate analysis. *CDKN2A/B* co-deletion was also the only factor associated with the occurrence of local progression using nominal logistic regression analysis (OR 5.33, 95% CI: 1.55-18.32, *P* = 0.008) (**Table 2 in the Supplement**).

**Table 2.** Univariate and Multivariate Cox Proportional Hazards Analyses Examining Factors Associated with Time to Local CNS Progression

	Univariate		Multivariate	
	OR (95% CI)	*P*-value	OR (95% CI)	*P*-value
*CDKN2A/B* Co-del	4.69 (1.57-14.01)	0.006	4.07 (1.32-12.54)	0.014
Age	1.05 (0.15-9.66)	0.96		
Male (vs female)	0.60 (0.19-1.96)	0.40		
Preop KPS	0.2 (0.03-2.2)	0.19		
Minority	1.29 (0.40-4.21)	0.67		
Primary cancer		0.84		
NSCLC	Reference			
Breast	1.73 (0.35-8.59)			
Melanoma	0.73 (0.12-4.39)			
GI	1.73 (0.29-10.38)			
Gyn	2.86 (0.48-17.15)			
RCC	2.68 (0.28-26.03)			
Other	* (*)			
No. total brain mets at surgery	0.016 (6.3e-7-4.54)	0.29		
Tumor volume	3.94 (0.38-25.38)	0.19		
Cystic	0.82 (0.18-3.71)	0.80		
Intratumoral hemorrhage	0.39 (0.12-1.28)	0.12		
Time from BM diagnosis to surgery	9.22 (0.68-63.58)	0.046	5.06 (0.31-43.25)	0.19
Presence of extracranial disease	0.50 (0.17-1.50)	0.22		
Side		0.18		
Right	Reference			
Left	2.22 (0.67-7.37)			
Midline	6.98 (0.73-66.61)			
Location		0.54		
Frontal	Reference			
Parietal	2.60 (0.47-14.24)			
Temporal	1.82 (0.26-12.95)			
Occipital	4.78 (0.80-28.69)			
Cerebellum	2.68 (0.37-19.27)			
GTR (vs STR)	1.19 (0.26-5.38)	0.82		
Prior intracranial radiotherapy	2.65 (0.81-8.63)	0.11		
Postoperative radiotherapy type		0.94		
Local RT	Reference			
WBRT	* (*)			
None	0.69 (0.09-5.32)			
Postop checkpoint inhibitor	0.7 (0.2-2.3)	0.54		
Postop targeted inhibitor	1.4 (0.5-4.1)	0.56		

Abbreviations: KPS, Karnofsky Performance Scale; NSCLC, non-small cell lung cancer; GI, gastrointestinal; Gyn, gynecologic; RCC, renal cell carcinoma; GTR, gross total resection; STR, subtotal resection; CNS, central nervous system; RT, radiation therapy; WBRT, whole brain radiation therapy.

^*^No events for statistical analysis.

*CDKN2A/B* co-deletion was also significantly associated with decreased time to distant CNS recurrence (**Figure 2B**, CDKN2A/B co-del vs no del: 4.2 vs 14.1 mo, log-rank *P* = 0.03). Univariate and multivariate Cox proportional hazard analyses were performed examining factors associated with distant CNS recurrence (**Table 3**). On univariate analysis, *CDKN2A/B* co-deletion, number of brain metastases present at surgery, tumor volume, increased time from brain met diagnosis to surgery, and prior intracranial radiation were associated with decreased time to distant progression. On multivariate analysis, *CDKN2A/B* co-deletion (HR 2.28, 95% CI 111-4.69, *P* = 0.025), number of total brain metastases at surgery (HR 18.18, 95% CI 2.48-100.56, *P* = 0.0017), and tumor volume (HR 5.71, 95% CI 1.32-20.99, *P* = 0.013) were associated with decreased time to distant CNS recurrence remote from the resection cavity.

**Table 3.** Univariate and Multivariate Cox Proportional Hazards Analyses Examining Factors Associated with Time to Distant CNS Progression

	Univariate		Multivariate	
	OR (95% CI)	*P*-value	OR (95% CI)	*P*-value
*CDKN2A/B* Co-del	2.05 (1.06-3.96)	0.033	2.28 (1.11-4.69)	0.025
Age	1.20 (0.40-3.94)	0.76		
Male (vs female)	1.51 (0.82-2.77)	0.18		
Preop KPS	0.8 (0.2-2.7)	0.69		
Minority	0.99 (0.50-1.93)	0.97		
Primary cancer		0.21		
NSCLC	Ref			
Breast	1.48 (0.54-4.10)			
Melanoma	2.89 (1.22-6.83)			
GI	1.56 (0.47-5.19)			
Gyn	1.82 (0.48-6.88)			
RCC	0.96 (0.12-7.66)			
Other	0.93 (0.28-3.09)			
No. total brain mets at surgery	12.31 (1.57-66.12)	0.0075	18.18 (2.48-100.56)	0.0017
Tumor volume	3.64 (0.93-11.81)	0.044	5.71 (1.32-20.99)	0.013
Cystic	0.36 (0.13-1.00)	0.05	0.37 (0.13-1.04)	0.06
Hemorrhage	0.84 (0.45-1.54)	0.57		
Time from BM diagnosis to surgery	6.02 (1.14-23.93)	0.019	2.52 (0.13-31.12)	0.51
Presence of extracranial disease	0.67 (0.36-1.23)	0.20		
Side		0.90		
Right	Ref			
Left	1.13 (0.61-2.10)			
Midline	0.85 (0.11-6.44)			
Location		0.68		
Frontal	Ref			
Parietal	1.58 (0.71-3.53)			
Temporal	1.00 (0.39-2.54)			
Occipital	1.58 (0.59-4.23)			
Cerebellum	0.92 (0.32-2.62)			
GTR (vs STR)	1.82 (0.71-4.63)	0.21		
Prior intracranial radiotherapy	2.03 (1.02-4.05)	0.045	1.42 (0.42-4.76)	0.57
Postoperative radiotherapy type		0.90		
Local RT	Ref			
WBRT	* (*)			
None	0.79 (0.28-2.21)			
Postop checkpoint inhibitor	1.2 (0.6-2.2)	0.60		
Postop targeted inhibitor	0.93 (0.5-1.7)	0.80		

Abbreviations: KPS, Karnofsky Performance Scale; NSCLC, non-small cell lung cancer; GI, gastrointestinal; Gyn, gynecologic; RCC, renal cell carcinoma; GTR, gross total resection; STR, subtotal resection; CNS, central nervous system; RT, radiation therapy; WBRT, whole brain radiation therapy.

^*^No events for statistical analysis.

Clinical and treatment variables based on *CDKN2A/B* co-deletion status were then analyzed, and only exposure to prior intracranial radiation before surgery was associated with *CDKN2A/B* co-deletion (**Table 4**). To ensure that survival bias did not account for increased risk of rapid local or distant recurrence, survival from surgery was compared based on *CDKN2A/B* co-deletion status which was not found to be different (*CDKN2A/B* co-del vs no del: 13.6 mo vs not reached, log-rank *P* = 0.13). Median follow-up was also not different between groups (*CDKN2A/B* co-del vs no del: 11.5 vs 13.3 mo, *P* = 0.55).

**Table 4.** Factors Associated with *CDKN2A/B* Co-deletion

	+*CDKN2A/B* co-del	−*CDKN2A/B* co-del	*P*-value
Age	64.8 ± 3.2	62.7 ± 1.7	0.58
Male	9 (42.9%)	32 (45.7%)	0.82
Female	12 (57.1%)	38 (54.3%)	
Preop KPS	74.3 ± 2.5	77.7 ± 1.4	0.24
Minority	6 (28.6%)	19 (27.1%)	0.90
Non-minority	15 (71.4%)	51 (72.9%)	
Primary cancer			0.13
NSCLC	9 (42.9%)	13 (18.6%)	
Breast	2 (9.5%)	13 (18.6%)	
Melanoma	6 (28.6%)	16 (22.9%)	
GI	3 (14.3%)	9 (12.9%)	
Gyn	0 (0%)	4 (5.7%)	
RCC	1 (4.8%)	3 (4.3%)	
Other	0 (0%)	12 (17.1%)	
No. total brain mets at surgery	2.95 ± 0.9	3.33 ± 0.5	0.71
Tumor volume	18.3 ± 4.6	22.5 ± 2.5	0.42
Cystic	4 (19.1%)	14 (20%)	0.92
Non-cystic	17 (80.9%)	56 (80%)	
Hemorrhage	11 (52.4%)	36 (51.4%)	0.94
No hemorrhage	10 (47.6%)	34 (48.6%)	
Time from BM diagnosis to surgery	6.0 ± 1.7	2.6 ± 0.9	0.08
Presence of extracranial disease	14 (66.7%)	46 (65.7%)	0.94
Absence of extracranial disease	7 (33.3%)	24 (34.3%)	
Side			0.59
Left	11 (52.4%)	32 (45.7%)	
Right	10 (47.6%)	35 (50%)	
Midline	0 (0%)	3 (4.3%)	
Location			0.64
Frontal	7 (33.3%)	19 (27.1%)	
Parietal	4 (19.1%)	20 (28.6%)	
Temporal	4 (19.1%)	12 (17.1%)	
Occipital	4 (19.1%)	7 (10%)	
Cerebellum	2 (9.5%)	12 (17.1%)	
EOR			0.60
GTR	17 (80.9%)	60 (85.7%)	
STR	4 (19.1%)	10 (14.3%)	
Prior intracranial radiotherapy			0.0083
Yes	8 (38.1%)	8 (11.4%)	
No	13 (61.9%)	62 (88.6%)	
Postoperative radiotherapy type			0.19
Local RT	16 (76.2%)	58 (82.9%)	
WBRT	2 (9.5%)	1 (1.4%)	
None	3 (14.3%)	11 (15.7%)	
Length of follow-up	11.5 ± 2.7	13.3 ± 1.5	0.55

Abbreviations: KPS, Karnofsky Performance Scale; NSCLC, non-small cell lung cancer; GI, gastrointestinal; Gyn, gynecologic; RCC, renal cell carcinoma; GTR, gross total resection; STR, subtotal resection; CNS, central nervous system; RT, radiation therapy; WBRT, whole brain radiation therapy.

## Discussion

In the era of precision medicine, identification of genomic alterations relevant to brain metastases is paramount to effectively target disease in this location. Indeed, genomic alterations may be site-specific, as has been demonstrated in numerous studies comparing various metastatic sites,^16–18^ and thus, obtaining tissue to guide further systemic therapy may become increasingly important as patient prognosis improves with better control of extracranial disease.

Surgery has been an important means for achieving local control of brain metastatic disease, especially, for patients with a single lesion or oligometastases with a dominant lesion with significant mass effect. In addition to local control, surgery offers the opportunity to obtain tissue to identify genetic alterations that may be targetable with systemic therapy or predictive of outcomes. Despite its role in brain metastasis management, there is a paucity of data examining the impact of genetic alteration observed with surgical outcomes such as local CNS recurrence.

In the present study, we examined gene mutations across 91 brain metastases derived from a range of primary cancer types to (a) assess common alterations across brain metastases and (b) identify mutations associated with local and distant CNS recurrence, markers of a more aggressive oncologic phenotype. *CDKN2A* and *CDKN2B* deletions were seen in 37% and 23% of samples, respectively, and homozygous co-deletion was seen in 23.1% of specimens. Furthermore, *CDKN2A/B* co-deletion was seen across multiple cancer types including NSCLC, melanoma, breast, gastrointestinal, and renal cell cancers. Although there was no association with survival after surgery, *CDKN2A/B* co-deletion was significantly associated with both local and distant CNS recurrence even when factoring in other patient and treatment factors suggesting a potential biologic role in CNS seeding and progression. To our knowledge, this is the first time, this genetic alteration has been reported to be associated with surgical outcomes after brain metastasis resection.

*CDKN2A* (i.e. Cyclin-dependent kinase inhibitor 2A) and *CDKN2B* (i.e. Cyclin-dependent kinase inhibitor 2B) are tumor suppressor genes located on 9p21 that encode for p16 and p15, respectively. These cell cycle checkpoint proteins interact with CDK4 and CDK6 to inhibit cell cycle progression. Co-deletion of these two genes has been associated with aggressive phenotypes in other oncologic settings including intracranial glioma and meningioma. In fact, the presence of homozygous *CDKN2A/B* deletion is now a diagnostic criterion for Astrocytoma WHO grade 4 in the new WHO 2021 classification system of CNS tumors.^19^ Furthermore, the cell cycle regulatory axis involving CDK4 and 6 is potentially targetable with new inhibitors such as ribociclib, palbociclib, and abemaciclib.^20^ These inhibitors may act to replace the lost inhibition seen with *CDKN2A/B* co-deletion.

Prior reports have demonstrated that *CDKN2A* is frequently altered across brain metastases from several cancer types. For example, Huang and colleagues found that *CDKN2A* and *CDKN2B* were frequently altered in NSCLC brain metastases,^21^ and in another study by the same group, *CDKN2A* and *CDKN2B* were also frequently altered in breast cancer brain metastases.^22^ Prior work has also suggested that *CDKN2A* deletion in melanoma patients is associated with an increased risk of developing brain metastases.^18,23,24^ Finally, *CDKN2A* genomic alterations have been identified with increased frequency in the cerebrospinal fluid of patients with leptomeningeal metastases.^25,26^ Indeed, genomic alterations predicting postoperative leptomeningeal disease after resection of brain metastases is an active area of investigation by our group.

### Limitations

Due to the retrospective nature of the study, there are some limitations including recall bias and heterogeneity in management during a patient’s oncologic course. We could only evaluate patients that had adequate documentation of clinical details with available imaging and genomic analysis of tumors.

## Conclusions

In this single-center study, we identify common genomic alterations in several genes across cancer types including *TP53* (64% of specimens), *CDKN2A* (37%), *TERT* (29%), *CDKN2B* (23%), *NF1* (14%), *KRAS* (14%), and *PTEN* (13%). *CDKN2A/B* co-deletion was seen across multiple cancer types and was associated with both local recurrence and more rapid distant CNS recurrences after surgical resection of brain metastases. Additional work is needed to determine whether therapies targeting pathways upregulated in brain metastases with *CDKN2A/B* alterations improve outcomes in these patients by preventing recurrence.

## Supplementary Material

vdad007_suppl_Supplementary_Figure_S1

vdad007_suppl_Supplementary_Tables
