# Supplementary figures and images for: CDKN2A/B co-deletion is associated with increased risk of local and distant intracranial recurrence after surgical resection of brain metastases
Source: Neurooncol Adv. 2023 Jan 28;5(1):vdad007. doi: 10.1093/noajnl/vdad007 (PMC10007908; doi:10.1093/noajnl/vdad007)

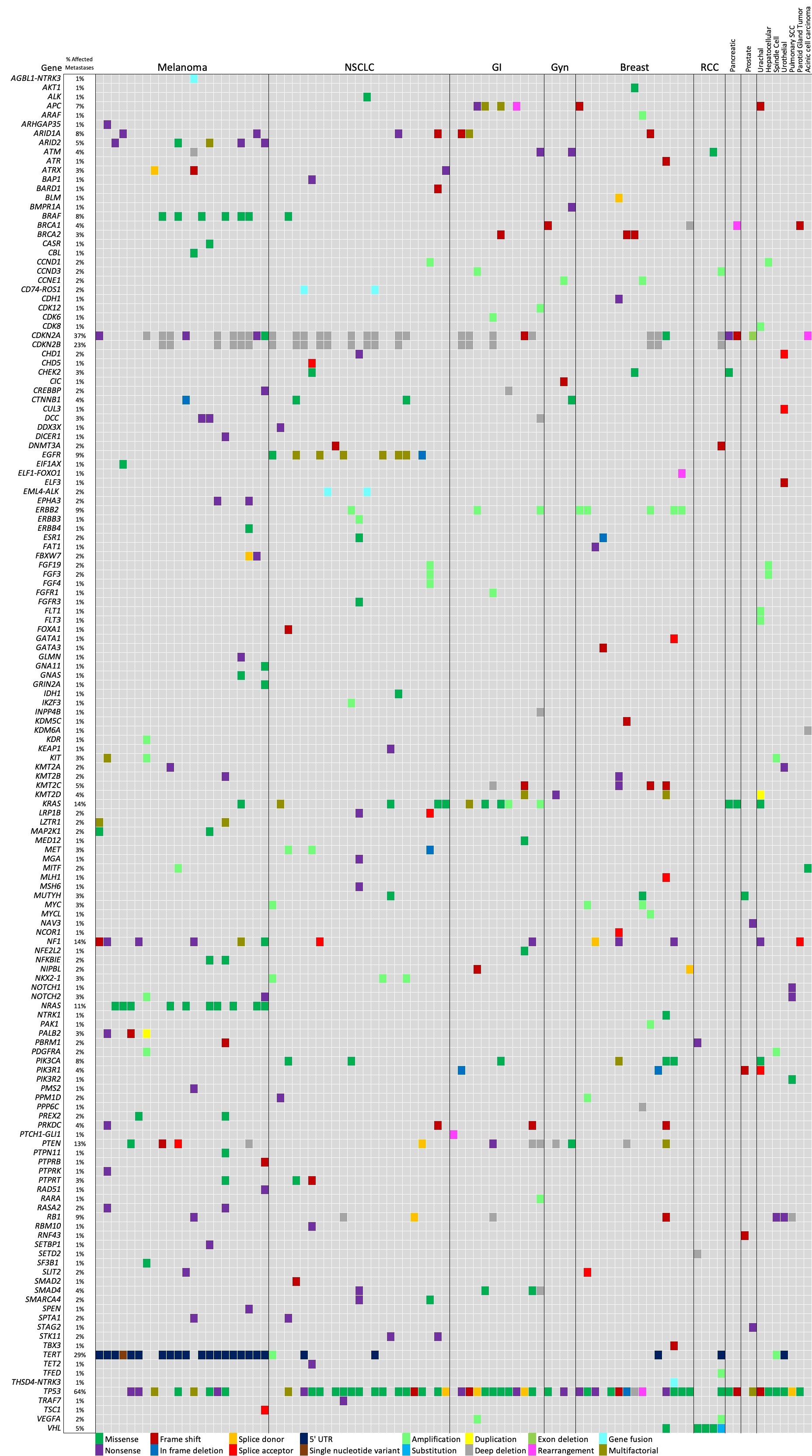

Supplement: vdad007_suppl_Supplementary_Figure_S1 [file vdad007_suppl_supplementary_figure_s1.jpeg]
