# Supplementary material for: CDKN2A/B co-deletion is associated with increased risk of local and distant intracranial recurrence after surgical resection of brain metastases
Source: Neurooncol Adv. 2023 Jan 28;5(1):vdad007. doi: 10.1093/noajnl/vdad007 (PMC10007908; doi:10.1093/noajnl/vdad007)
Supplement: vdad007_suppl_Supplementary_Tables [file vdad007_suppl_supplementary_tables.docx]

**Supplemental Table 1.** Genomic alterations identified leading to changes in targeted inhibitor treatment

| **Age/Sex** | **Primary Cancer** | **Genomic Alterations** | **Postop Targeted Inhibitor Trialed** |
| --- | --- | --- | --- |
| 66M | Melanoma | - NRAS - TERT - ARID1A - EIF1AX | Cobimetinib (MEK Inhibitor) |
| 53F | Melanoma | - BRCA1 - CDKN2A - KIT - PDGFRA - KDR - SF381 - NOTCH2 | Imatinib (KIT inhibitor), Binimetinib (MEK inhibitor) |
| 69F | NSCLC | - CDKN2A - CDKN2B - EGFR - MYC - . NKX2-1 - TERT | Osimertinib (EGFR inhibitor) |
| 67F | NSCLC | - CDKN2A - CDKN2B - CTNNB1 - EGFR - PTPRT - SMAD2 | Osimertinib (EGFR inhibitor) |
| 72M | NSCLC | - CDKN2A - CDKN2B - EGFR - TP53 - NF1 | Osimertinib (EGFR inhibitor) |
| 33M | Melanoma | - BRAF - CDKN2A - CDKN2B - PTEN - TERT | Dabrafenib (BRAF inhibitor), Trametinib (MEK inhibitor) |
| 46F | Breast | - BRCA2 - PTEN - KDM5C - TP53 | Olaparib (PARP inhibitor) |
| 76F | NSCLC | - EGFR - RB1 - TP53 - TRAF7 | Osimertinib (EGFR inhibitor) |
| 70M | Melanoma | - CDKN2A - CDKN2B - NRAS - TERT - KMT2A | Binimetinib (MEK Inhibitor) |
| 75F | Serous Carcinoma | - BRCA1 - TP53 | Niraparib (PARP Inhibitor) |
| 49M | NSCLC | - ALK - EML4-ALK Fusion - CDKN2A - CDKN2B | Lorlatinib (ALK Inhibitor) |
| 52F | NSCLC | - EGFR - NKX2-1 - TP53 | Osimertinib (EGFR inhibitor) |
| 64F | NSCLC | - CDKN2A - CDKN2B - EGFR - IDH1 - TP53 - ARID1A | Osimertinib (EGFR inhibitor) |
| 73M | Melanoma | - CDKN2A - CDKN2B - NRAS - TERT | Trametinib (MEK Inhibitor) |
| 35F | Breast | - KAT1 - BRCA2 - TP53 - CHEK2 | Olaparib (PARP inhibitor) |
| 65M | NSCLC | - CDKN2A - CTNNB1 - EGFR - NKX2-1 - TP53 | Osimertinib (EGFR inhibitor) |
| 70F | Melanoma | - BRAF - CDKN2A - CDKN2B - PTEN - TERT - EPHA3 - ERBB4 - FBXW7 - SPEN | Encorafenib (BRAF inhibitor) |
| 55F | Breast Adenocarcinoma | - ERBB2 - TPO53 - APC | Tucatinib and Trastuzumab (HER2 receptor inhibitor) |
| 72M | Esophageal Adenocarcinoma | - HER2 + on IHC - CDKN2A - NF1 - PTEN - SMAD4 - TP53 - PRKDC | Trastuzumab (HER2 receptor inhibitor) |
| 84F | NSCLC | - CCND1 - FGF19 - FGF4 - FGF3 - MET - LRP1B - SMARCA4 | Capmatinib (c-Met inhibitor) |
| 53F | NSCLC | - EGFR - PTEN - TP53 | Osimertinib (EGFR inhibitor) |
| 60F | Acinic Cell Carcinoma | - CDKN2A - KDM6A - MITF | Palbociclib (CDK inhibitor) |

**Supplemental Table 2.** Univariate nominal logistic regression analyses examining factors associated with local CNS progression.

|  | **Univariate** | |
| --- | --- | --- |
|  | **OR (95% CI)** | **p-value** |
| ***CDKN2A/B* co-del** | 5.33 (1.55-18.32) | 0.008 |
| **Age** | 0.99 (0.95-1.03) | 0.63 |
| **Male (vs Female)** | 0.49 (0.14-1.73) | 0.27 |
| **Preop KPS** | 0.2 (0.02-2.4) | 0.20 |
| **Minority** | 1.21 (0.34-4.34) | 0.77 |
| **Primary Cancer**  ***NSCLC***  ***Breast***  ***Melanoma***  ***GI***  ***Gyn***  ***RCC***  ***Other*** | Ref  1.58 (0.27-9.17)  0.63 (0.10-4.22)  1.27 (0.18-8.87)  6.33 (0.63-63.64)  2.11 (0.16-27.58)  * (*) | 0.69 |
| **No. Total Brain Mets at Surgery** | 0.81 (0.58-1.13) | 0.22 |
| **Tumor Volume** | 1.03 (0.99-1.04) | 0.10 |
| **Cystic** | 0.70 (0.14-3.50) | 0.67 |
| **Intratumoral hemorrhage** | 0.36 (0.10-1.27) | 0.11 |
| **Time from BM diagnosis to Surgery** | 1.04 (0.98-1.11) | 0.17 |
| **Presence of Extracranial Disease** | 0.38 (0.12-1.25) | 0.11 |
| **Side**  ***Right***  ***Left***  ***Midline*** | Ref  2.34 (0.65-8.44)  5.13 (0.38-69.75) | 0.30 |
| **Location**  ***Frontal***  ***Parietal***  ***Temporal***  ***Occipital***  ***Cerebellum*** | Ref  2.4 (0.40-14.49)  1.71 (0.22-13.56)  4.5 (0.63-31.95)  2 (0.25-15.99) | 0.66 |
| **GTR vs STR** | 1 (0.20-5.09) | 1.00 |
| **Prior Intracranial Radiotherapy** | 2.44 (0.65-9.23) | 0.19 |
| **Postoperative Radiotherapy Type**  ***Local RT***  ***WBRT***  ***None*** | Ref  * (*)  0.40 (0.05-3.33) | 0.70 |

NSCLC: non-small cell lung cancer; GI: gastrointestinal; Gyn: gynecologic; RCC: renal cell carcinoma; GTR: gross total resection; STR: subtotal resection; CNS: central nervous system; RT: radiation therapy; WBRT: whole brain radiation therapy

* No events for statistical analysis
